# Identification of early predictors for infected necrosis in acute pancreatitis

**DOI:** 10.1186/s12876-022-02490-9

**Published:** 2022-09-03

**Authors:** Mats L. Wiese, Steffi Urban, Sabrina von Rheinbaben, Fabian Frost, Matthias Sendler, Frank Ulrich Weiss, Robin Bülow, Marie-Luise Kromrey, Quang Trung Tran, Markus M. Lerch, Birgit Schauer, Ali A. Aghdassi

**Affiliations:** 1 Department of Medicine A, University Medicine Greifswald, Ferdinand-Sauerbruch-Straße, 17475 Greifswald, Germany; 2 Institute of Diagnostic Radiology and Neuroradiology, University Medicine Greifswald, Greifswald, Germany; 3 Department of Internal Medicine, University of Medicine and Pharmacy, Hue University, Hue City, Vietnam; 4 Ludwigs-Maximilians University Munich, Munich, Germany; 5 Institute for Community Medicine, University Medicine Greifswald, Greifswald, Germany

**Keywords:** Acute pancreatitis, Infected necrosis, Prediction, Multivariate model, ROC analysis

## Abstract

**Background:**

In acute pancreatitis, secondary infection of pancreatic necrosis is a complication that mostly necessitates interventional therapy. A reliable prediction of infected necrotizing pancreatitis would enable an early identification of patients at risk, which however, is not possible yet.

**Methods:**

This study aims to identify parameters that are useful for the prediction of infected necrosis and to develop a prediction model for early detection. We conducted a retrospective analysis from the hospital information and reimbursement data system and screened 705 patients hospitalized with diagnosis of acute pancreatitis who underwent contrast-enhanced computed tomography and additional diagnostic puncture or drainage of necrotic collections. Both clinical and laboratory parameters were analyzed for an association with a microbiologically confirmed infected pancreatic necrosis. A prediction model was developed using a logistic regression analysis with stepwise inclusion of significant variables. The model quality was tested by receiver operating characteristics analysis and compared to single parameters and APACHE II score.

**Results:**

We identified a total of 89 patients with necrotizing pancreatitis, diagnosed by computed tomography, who additionally received biopsy or drainage. Out of these, 59 individuals had an infected necrosis. Eleven parameters showed a significant association with an infection including C-reactive protein, albumin, creatinine, and alcoholic etiology, which were independent variables in a predictive model. This model showed an area under the curve of 0.819, a sensitivity of 0.692 (95%-CI [0.547–0.809]), and a specificity of 0.840 (95%-CI [0.631–0.947]), outperforming single laboratory markers and APACHE II score. Even in cases of missing values predictability was reliable.

**Conclusion:**

A model consisting of a few single blood parameters and etiology of pancreatitis might help for differentiation between infected and non-infected pancreatic necrosis and assist medical therapy in acute necrotizing pancreatitis.

**Supplementary Information:**

The online version contains supplementary material available at 10.1186/s12876-022-02490-9.

## Background

Acute pancreatitis is the most frequent non-malignant gastroenterological disorder leading to hospitalization in Western countries. It accounts for almost 280,000 hospital admissions in the US [1] and around 55,000 in Germany per year [2]. While the majority of patients suffers from a mild disease with an uneventful recovery, there is a severe course of acute pancreatitis in 10 to 15% of cases leading to organ or even multi-organ failure, necessity for intensive care therapy and a high mortality [3]. Besides organ failure, approximately 5 to 20% of patients develop necrotizing pancreatitis, involving the pancreas, the surrounding fatty tissue or both [4]. Necroses may cause further local complications such as compression of adjacent organs, increase of intraabdominal pressure or gastric outlet obstruction. Secondary infection of the necrotic tissue is a severe condition with increased morbidity and mortality [5] requiring antibiotic treatment or even invasive interventions [6, 7].

Diagnosis of an infected necrosis is still challenging and often it needs to be confirmed ultimately by microbiological analysis after fine-needle aspiration or even drainage, measures that have to be carried out judiciously because they also encompass a periprocedural risk [8]. Established multiparameter scores such as the Acute Physiology and Chronic Health Evaluation II (APACHE II) score [9] and the Ranson Score [10] have been used for grading disease severity and prediction of mortality. However, they are cumbersome to calculate as they need a large number of parameters requiring different time points and their predictive accuracy for infected necrosis is unclear. Several routine laboratory parameters, for instance, markers for inflammation, kidney function or hematocrit [11], have been attempted for accurate prediction of severe acute pancreatitis, development of necrosis and mortality. Despite promising potential, these measurements have to be repeated at later time points and their usability is limited when using every single parameter alone. So far predictive factors of infected pancreatic necrosis, allowing the initiation of an early and preemptive therapy to improve the outcome of acute necrotizing pancreatitis, have not been established.

## Methods

### Study design and patient selection

This study aimed to identify parameters associated with infected pancreatic necrosis that are already assessable in early disease, ideally at admission to hospital, and, in a second step, to derive a predictive composite metric from these parameters. In this retrospective single center study, we investigated patients with acute necrotizing pancreatitis who underwent either aspiration or drainage of a pancreatic necrotic collection. Data were retrieved from the hospital information and reimbursement data system of the University Medicine Greifswald, a tertiary medical center in northeast Germany, between January 2009 and December 2019. Diagnosis of acute pancreatitis was established by fulfilment of at least two of the following three criteria: a) abdominal pain clinically consistent with acute pancreatitis, b) elevation of serum lipase of at least three times of upper limit of normal (ULN), and c) typical signs of acute pancreatitis in imaging [12].

Potentially eligible patients were identified by the combination of a diagnosis of acute pancreatitis according to ICD-10 (K85.XX) and a therapeutic medical procedure encoded by the German procedure classification system (OPS), consisting of a contrast enhanced abdominal CT-scan (OPS 3-225) combined with endoscopic-guided fine needle aspiration (OPS 1-447, OPS 5-529) or percutaneous drainage (OPS 8-146). Presence of pancreatic or peripancreatic necroses were confirmed by two radiologists (RB, MLK) experienced in gastrointestinal imaging. Prior to data retrieval the study was approved by the local institutional review board of the University of Greifswald (registration no. BB 138/19) that waived requirement for patient’s informed consent.

### Patient’s medical history

For each patient data on age, sex, etiology of acute pancreatitis, history of alcohol and nicotine consumption was extracted from medical records. Vital and blood parameters as well as the APACHE II score [9] were recorded at the time point of admission to our institution. Relevant co-existing disorders were subsumed in the Charlson Comorbidity Index (CCI) [13]. Previous antibiotic treatment before intervention of the necrosis was noted for every patient. Length of hospital stay as well as the requirement of both intensive and intermediate care treatment were recorded. For patients being transferred from another hospital, length of the previous stay was included in the calculation of total hospital stay.

### Diagnosis of infected necrosis, systemic complications, and mortality

Patients with suspected pancreatic necrosis and clinical suspicion of an infection underwent either endoscopic ultrasound-guided fine needle aspiration or direct drainage of the necrotic cavity, which was performed by a transmural or percutaneous approach. Infection of pancreatic necrosis was diagnosed microbiologically by Gram staining and culture of biopsy material for bacteria or fungi. In case of multiple interventions, pancreatic necrosis was classified as infected when there were signs of an infection in at least one sample.

Systemic organ complications included cardiovascular, respiratory, or renal failure. Cardiovascular failure was defined as a decrease of systolic or mean arterial pressure to less than 90 mmHg or 60 mmHg, respectively, irresponsive of fluid administration [14]. Respiratory failure was considered in case of need for mechanical ventilation and renal failure as an increase of serum creatinine by at least 1.5 × ULN from baseline according to the Kidney Disease Improving Global Outcomes classification [15]. In addition, mortality of patients due to acute pancreatitis or its complications was recorded.

### Statistical analysis

Data were analyzed using SPSS Statistics 27 (IBM, Ehningen, Germany). To test for differences between groups, two-tailored t-test or Kruskal–Wallis test were used for normally or non-normally distributed continuous variables, respectively. Differences in categorical variables were assessed by χ^2^- or Fisher’s exact test, in case of cells with an expected frequency of less than five. The association of laboratory parameters with infected pancreatic necrosis was tested by applying a binary logistic regression model.

For development of a prediction model for infected necrosis we performed stepwise logistic regression analyses. A forward stepwise procedure was used to select the independent variables with highest predictive value for inclusion in the final multivariable model. Variables initially considered for inclusion comprised routine blood parameters, vital parameters, comorbidities, medication, etiology of acute pancreatitis, age, sex, and BMI. Variables significantly associated with infected necrosis were added to the model in a stepwise manner according to their predictive value, indicated by pseudo R^2^ values, i.e. Nagelkerke’s R^2^ and Cox & Snell R^2^, until no further improvement of the model was achieved. Receiver operating characteristic (ROC) analysis was then performed to compare predictive performance of the model with single parameters. To identify the optimal cut-off value, Youden’s J statistic [16] was calculated. *P*-values of < 0.05 and < 0.001 were considered to be significant and highly significant, respectively.

## Results

### Patient selection and characteristics

**Fig. 1:**
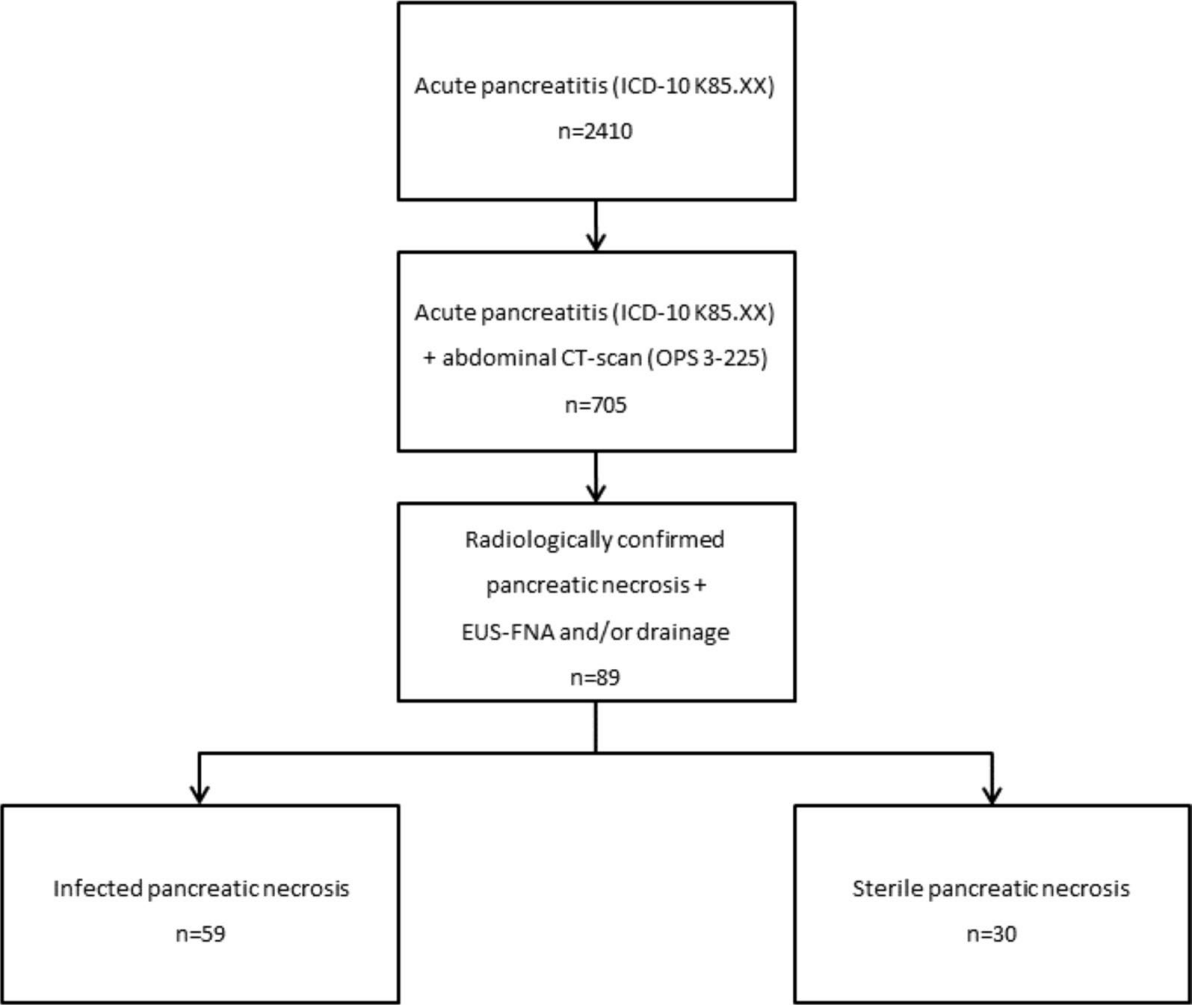
Flowchart describing the patient identification and selection process

Between 2009 and 2019 a total of 2,410 patients with diagnosis of acute pancreatitis (K85.XX) were admitted to our hospital. Among them 705 received an abdominal CT-scan (OPS 3-225) and in 89 patients there was either an acute necrotic collection or walled-off necrosis that were treated by either fine needle aspiration (OPS 1-447), endoscopic or percutaneous drainage (OPS 5-529). Only fine needle aspiration was performed in 14 patients, whereas 75 individuals underwent drainage therapy. In total, 59 subjects had an infected necrosis whereas no growth of bacteria or fungi was detected in the other 30 patients (Fig. 1). In the majority of patients with infected necroses (81.4%) diagnosis was established by the first intervention. Proof of microbial infection by the second or third intervention was given 13.6% and 5.1% of the cases. Patients with infected pancreatic necrosis did not differ from those with sterile necrosis regarding age, sex, BMI, smoking status, location of necrosis, CCI, as well as the prevalence of diabetes mellitus or exocrine insufficiency (Table1). Regarding etiology, patients with sterile necrosis were more likely to have acute on chronic pancreatitis (*p* = 0.028), although these numbers were rather low compared to other causes of acute pancreatitis. Alcoholic etiology tended to be more common in patients with infected necrosis (*p* = 0.051). APACHE II score at admission was significantly higher in infected than in sterile necrosis (*p* = 0.001). Regarding the size of pancreatic necrosis we classified their extent into areas of < 30%, 30–50%, and > 50% as described by Balthazar et al. [17] (Additional file 1: Table S1). For all three categories the distribution of the necrotic areas was similar showing no differences between patients with sterile and infected necroses (*p* = 0.426).

**Table 1:** Characterization of the patient cohort

	Infected necrosis (n = 59)	Sterile necrosis (n = 30)	*p*-value^a^
Mean age (± SD), years	59.37 (± 15.05)	55.97 (± 15.26)	0.318
Sex (male), n (%)	48 (81.4)	24 (80.0)	0.878
Median BMI (IQR)^b^, kg/m^2^	26.00 (3.80)	25.00 (5.00)	0.271
Smoking, n (%)	23 (54.8)	11 (55.0)	0.986
Etiology of acute pancreatitis, n (%)			
Alcohol	24 (40.7)	6 (20.0)	0.051
Biliary	15 (25.4)	12 (40.0)	0.157
Acute on chronic pancreatitis	2 (3.4)	5 (16.7)	0.028
Post ERCP	2 (3.)	1 (3.3)	0.989
Other, including idiopathic	16 (27.1)	6 (20.00)	0.462
Localization of necrosis, n (%)			
Pancreatic head	31 (52.5)	11 (36.7)	0.156
Pancreatic body	35 (59.3)	16 (53.3)	0.589
Pancreatic tail	36 (61.0)	23 (76.7)	0.140
Peripancreatic	11 (18.6)	2 (6.7)	0.130
Prior antibiotic therapy	25 (42.4)	3 (10.0)	0.002
Median APACHE-2 Score (IQR)^c^	10.00 (9.00)	5.00 (5.00)	0.001
Median Charlson Comorbidity Index (IQR)	4.00 (3.00)	2.00 (4.00)	0.099
Diabetes mellitus, n (%)	18 (30.5)	7 (23.3)	0.476
Exocrine insufficiency, n (%)	14 (23.7)	3 (10.0)	0.119

^a^Significant differences between groups were tested using two-tailed t-test for normally distributed continuous variables, Kruskal–Wallis test for non-normally distributed continuous variables, and χ^2^- test or Fisher’s exact test for categorical variables

^b^Infected necrosis (n = 40), sterile necrosis (n = 23)

^c^Infected necrosis (n = 50), sterile necrosis (n = 28)

### Microbial composition of infected necrosis

In the majority of patients with infected pancreatic necrosis multiple microorganisms were detected. Gram-positive bacteria were found in 43 (72.88%) of individuals, among them *Enterococcus faecium* was predominant. In 30 infected necroses gram-negative bacteria could be identified and the three most common bacteria were *Escherichia coli*, *Pseudomonas aeruginosa*, and *Klebsiella*, found in 9, 8, and 6 patients, respectively. Moreover, 26 infected necroses contained fungal pathogens, with *Candida albicans* as the most common species. Results are summarized in Additional file 2: Table S2.

### Association of infected necrosis with clinical outcome

A comparison of outcome parameters between patients with infected and sterile pancreatic necrosis is presented in Table 2. Patients with infected necrosis more frequently developed both renal and respiratory failure (*p* = 0.002 and *p* < 0.001, respectively). In addition, the percentage of patients requiring intensive care unit (ICU) or intermediate care (IMC) treatment was significantly higher in those with infected necrosis (*p* = 0.001 and 0.017, respectively). While, median length of hospital stay was almost twice as long in infected necrosis (54 vs. 28 days, *p* < 0.001), there was no significant difference in mortality between the two groups (*p* = 0.432).

**Table 2:** Outcome parameters in infected and sterile necrosis

	Infected necrosis(n = 59)	Sterile necrosis(n = 30)	*p*-value^a^
Respiratory failure [need for mechanical ventilation], n (%)	25 (42.4)	3 (10.0)	0.002
Cardiovascular failure [systolic blood pressure < 90 mmHg or mean arterial pressure < 60 mmHg], n (%)^b^	2 (3.4)	0 (0.0)	0.292
Renal failure [creatinine > 1.5 × ULN of baseline], n (%)	28 (47.5)	3 (10.0)	< 0.001
Requiring ICU treatment, n (%)	34 (57.6)	6 (20.0)	0.001
Requiring IMC, n (%)	41 (69.5)	13 (43.3)	0.017
Mortality, n (%)	5 (8.5)	1 (3.3)	0.432
Median Length of hospital stay, days (IQR)	54 (60)	28 (25)	< 0.001

^a^Significant differences between groups were tested using Kruskal–Wallis test for non-normally distributed continuous variables, and χ^2^- test or Fisher’s exact test for categorical variables

^b^Infected necrosis (n = 50), sterile necrosis (n = 27)

### Association of infected necrosis with blood parameters

Nine out of 20 blood parameters analyzed were significantly associated with infected pancreatic necrosis (Table 3). These comprised calcium, creatinine, urea, albumin, total leukocyte count, total bilirubin, C-reactive protein (CRP), prothrombin time, and lactate dehydrogenase. While most parameters were available in at least 90% of the patients, other parameters, not taken on a routine basis, e.g. interleukin-6 and procalcitonin were measured in less than 60%. The strongest associations with infected pancreatic necrosis were seen for creatinine (OR [95% CI] 1.019 [1.005–1.033], *p* < 0.001), CRP (OR [95% CI] 1.009 [1.004–1.014], *p* < 0.001), and albumin (OR [95% CI] 0.914 [0.861–0.970], *p* = 0.002).

**Table 3:** Association of blood parameters with infected pancreatic necrosis

	n	Odds ratio	95%-CI	Cox & snell R^2^	Nagelkerke’s R^2^
Sodium	89	0.999	0.911–1.095	0.000	0.000
Potassium	89	0.932	0.424–2.050	0.000	0.000
Calcium	88	0.130	0.016–1.047	0.054	0.075
Creatinine	89	1.019	1.005–1.033	0.162	0.225
Urea	88	1.190	1.040–1.363	0.107	0.149
Albumin	78	0.914	0.861–0.970	0.116	0.162
Total leukocyte count	89	1.094	1.013–1.181	0.067	0.093
Total thrombocyte count	89	0.998	0.996–1.001	0.016	0.023
Hematocrit	89	0.339	0.001–170.683	0.001	0.002
Lipase	82	1.000	0.997–1.003	0.000	0.000
Bilirubin	88	1.028	0.993–1.064	0.056	0.076
C-reactive protein	88	1.009	1.004–1.014	0.159	0.220
Procalcitonin	52	0.992	0.941–1.047	0.001	0.002
Interleukin 6	41	1.000	0.999–1.001	0.009	0.014
Prothrombin time	89	0.976	0.956–0.997	0.060	0.084
Total triglycerides	50	1.085	0.835–1.409	0.008	0.012
pH value	71	0.002	0.000–8.218	0.037	0.055
Lactate	71	1.138	0.831–1.558	0.015	0.022
Lactate dehydrogenase	63	1.238	1.015–1.510	0.108	0.155
Blood glucose	80	1.062	0.941–1.199	0.013	0.018

### Prediction model for infected necrosis

To develop a predictive model for an early detection of infected pancreatic necrosis, a multivariate analysis was performed. Details of the final prediction model are presented in Table 4. Besides creatinine, CRP, and albumin, the final model also included alcoholic etiology as a predictor. Cox & Snell R^2^ and Nagelkerke’s R^2^ values of 0.360 and 0.502, respectively, indicated good model fit. Iterations of model development including the complete list of parameters that were considered are provided as supplementary material (Additional file 3: Table S3).

**Table 4:** Multivariate logistic regression model for prediction of infected pancreatic necrosis

Predictor	Regression coefficient	Standard error	Wald *Χ*^*2*^	*p*-value	Odds ratio	95%-CI
Creatinine	0.026	0.010	6,478	0.011	1.026	1.006–1.047
Albumin	− 0.066	0.045	2.151	0.142	0.936	0.858–1.022
Alcoholic etiology	1.759	0.765	5.295	0.021	5.808	1.298–25.992
C-reactive protein	0.006	0.003	3.287	0.070	1.006	1.000–1.013
Constant	− 1.504	1.579	0.907	0.341	0.222	–

*Cox & Snell R*^*2*^*: 0.360Nagelkerke’s R*^*2*^*: 0.502*

### Model performance

**Fig. 2:**
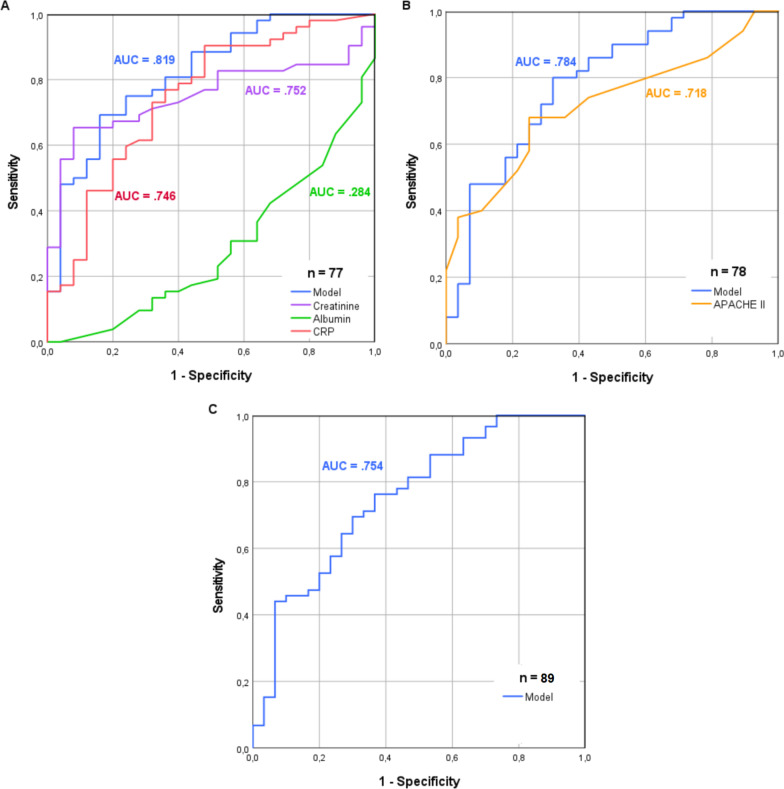
Receiver operating characteristic curves. Performance of the predictive model in comparison to **a** single laboratory parameters, **b** the APACHE II score, and **c** applied to the entire patient collective

In a next step, ROC curves were plotted to assess both the performance of each single laboratory result and a combination of the aforementioned parameters to predict the presence of an infected necrosis. The results of ROC analysis are shown in Fig. 2. With an AUC of 0.819 the prediction model achieved greater AUC than creatinine, CRP, or albumin, respectively (Fig. 2a) and also surpassed performance of the APACHE II score, a widely accepted assessment tool for disease severity and mortality (Fig. 2b). Besides, despite the unavailability of single parameters in 12 patients, the prediction model reached an AUC of 0.754 when applied to the entire patient collective (Fig. 2c). With a sensitivity of 0.692 (95%-CI [0.547–0.809]) and a specificity of 0.840 (95%-CI [0.631–0.947]) we identified a value of 0.25 as the ideal cut-off point.

## Discussion

Infected necrosis is a severe complication of acute pancreatitis that usually arises during the later phase of pancreatitis. In this study, we identified parameters associated with infection of necrosis in acute pancreatitis. Based on these findings, we developed a logistic regression model based on blood levels of creatinine, albumin, and CRP, as well as alcoholic etiology that predicts infection with higher accuracy than any individual laboratory parameter or the APACHE II score.

The parameters we finally included in our prediction model are coherent with existing literature on prediction of the course and complications in acute pancreatitis. For instance, CRP, an acute-phase reactant, has been shown repeatedly to predict severity of acute pancreatitis—although there has been debate about the optimal time point and cut-offs [18, 19]. Moreover, CRP had a good prognostic accuracy not only for severe acute pancreatitis but also pancreatic necrosis and in-hospital mortality [20]. Prognostic value has also been found specifically regarding development of secondary infections in acute pancreatitis [21].

Likewise, regarding creatinine, there is evidence that elevated levels in early disease can predict pancreatic necrosis [22, 23]. It is conclusive that creatinine also predicts secondary infection of pancreatic necrosis as it indicates impaired renal function and potential subsequent renal failure, which we found to be associated with infected necrosis.

Inclusion of albumin in the final prediction model was not an unexpected finding. Serum albumin has been found predictive of persistent organ failure in acute pancreatitis in multiple studies [24, 25]; and in our analysis it was linked to infected pancreatic necrosis as well. Being independently associated with both inflammation and a compromised nutritional status in acute conditions [26], hypoalbuminemia—by the very same mechanisms—could not only predispose to organ failure but also infection of pancreatic necrosis.

Although the role of etiology on course and progression of acute pancreatitis has been discussed controversially for a long time, recent findings support the relevance of alcoholic etiology for prediction of infected pancreatic necrosis. A recent meta-analysis found necrosis to be more common in alcoholic than biliary pancreatitis [27]—the two most common etiologies in acute pancreatitis. Additionally, evidence has accumulated from experimental studies that alcohol increases intestinal permeability and thus facilitates translocation of both bacteria and bacterial products [28] that could elicit infection of pancreatic necrosis.

A number of multiparameter predictors have been evaluated for prediction of adverse outcome in acute pancreatitis [29]. The APACHE II system is one of the most widely used severity scores for critically ill patients, which incorporates both markers of patient physiology recorded immediately or shortly after hospital admission and chronic comorbidity categories. Due to these known relations, we evaluated this score regarding a potential link to infected necrosis as well. There was an association of APACHE II score with infected pancreatic necrosis. However, our model outperformed it. Considering that the APACHE II score is not specific for acute pancreatitis and requires multiple items that in part are laborious to record, such as parameters for blood oxygenation, its usefulness for prediction of infected necrosis seems to be limited in clinical practice [28].

In an earlier study, Chen et al. [30] used a similar approach to develop a prediction model for infection of pancreatic necrosis. Their final model included different parameters than ours. However, these aberrant findings do not necessarily contradict our results. First, etiologies of acute pancreatitis differed in the two cohorts. We had more alcoholic than biliary pancreatitis, which was the most common cause apart from hyperlipidemia in the study by Chen and co-workers. In addition, the studies were conducted in two different countries and findings in Asian populations cannot be transferred unrestrainedly to Western populations and vice versa [31]. We also included patients presenting with acute on chronic pancreatitis, which were excluded in the other study. Further, we investigated a wider a range of clinical and laboratory parameters and included, for instance, albumin, which we found to be an independent predictor of infected necrosis.

One must also consider parameters that have been suggested as predictors of infected necrosis before but did not contribute to prediction in the current study. For instance, higher median procalcitonin (PCT) concentrations have been found in patients with infected necrosis and a complicated course of acute pancreatitis resulting in death [32]. In our patients, overall mortality was as low as 6.7% percent, which could explain why we did not find an association. Besides, earlier findings suggest that PCT is not a specific marker of infected necrosis as it is also elevated in septic patients without pancreatitis [33]. Moreover, it has been hypothesized that PCT levels in acute pancreatitis are elevated as part of the systemic inflammatory response and therefore not necessarily indicate infection [34].

Blood urea nitrogen (BUN) has been reported with alleged predictive value as a rise in blood urea nitrogen within 48 h was associated with a risk for the development of infected pancreatic necrosis [35]. Although we found an association between BUN and infected pancreatic necrosis as well, the association was weaker than with other parameters and inclusion of BUN did not further improve the prediction model. Besides analyzing BUN at a single time point, a high correlation with creatinine, another indicator of renal function and the strongest single predictor of infected necrosis in our study, could explain why BUN was not included in our final prediction model.

There are limitations to our study. These are partly owed to its retrospective and monocentric design, including incomplete patient data and blood values as well as assessment of blood parameters only at time of admission. Therefore, there is a residual chance that we missed relevant parameters, especially those that show a dynamic during the course of diseases. On the contrary, our results realistically reflect the situation in clinical practice. It can be cumbersome and costly to monitor the course of multiple, potentially not routine blood parameters over a longer time. Hence, our prediction model likely presents a more feasible approach. However, it needs to be emphasized that its predictive performance has not been validated prospectively so far. A prospective trial will be necessary to confirm the validity of our model developed from the retrospectively collected data. Another limitation of our analysis is that we also included patients transferred from external hospitals. This may include that treatment of acute pancreatitis at least during the early phase was not uniform in all cases because local expertise varies in smaller district hospitals. In addition, time between actual onset of pain and hospital admission could vary leading to an inhomogeneous patient cohort regarding stage of pancreatitis. Although only individuals with microbiologically proven infection were included in our study there is a risk of false positive or negative results even after microbiologic analysis of the necrotic material which have been reported in up to 15% and 25% of cases, respectively [36]. In addition, the number of actually infected necroses might be lower as secondary infections might occur not only after percutaneous but even after endoscopic guided drainages of pancreatic necrotic collections and repeated necrosectomies. For further clarification of microbial transmissions rates into drained necroses additional studies will be necessary. The putative low number of patients with sterile necrosis (n = 30) in this investigation resulted from the fact, that only individuals with proven negative results on microbial culture were selected, even after repeated biopsies. Due to the selection of patients who have undergone intervention we observed a larger proportion of individuals with infected necrosis than reported in previous studies [37]. Under some circumstances a primarily conservative therapeutic strategy based on solely antibiotic treatment and drainage only if unavoidable, can be as effective as an immediate drainage therapy in terms of mortality [38]. Because suspected infected necroses could not be captured by ICD-10 codes, we have potentially missed patients with infected pancreatic necrosis who neither underwent EUS-FNA nor drainage for our model. Last, some patients may have responded to prophylactic antibiotic treatment that was given empirically without prior microbial confirmation and therefore did not develop infected necrosis. Nevertheless, the chance that predictive performance of our model was hampered by such treatment response is rather low as an infected necrosis was detected in almost 90% of patients receiving antibiotics.

## Conclusions

We could develop a prediction model for identification of infected necrosis in acute pancreatitis. It might help to avoid overhasty interventions on pancreatic necrosis in situations when infections are suspected. Including only four parameters, already assessable in early disease, our model could facilitate clinical decision-making in treatment of acute pancreatitis. We therefore encourage use of this model in future prospective studies to validate its clinical relevance.

## Supplementary Information

**Additional file 1**. **Table S1.** Model development with complete list of parameters.

**Additional file 2**. **Table S2.** Microbial composition of infected necrosis (n = 59).

**Additional file 3**. **Table S3.** Model development with complete list of parameters.

## Data Availability

The datasets used and/or analysed during the current study are available from the corresponding author on reasonable request.

## Abbreviations

APACHE II: Acute physiology and chronic health evaluation
BUN: Blood urea nitrogen
CCI: Charlson comorbidity index
CI: Confidence interval
CRP: C-reactive protein
ICD-10: International classification of diseases-10
ICU: Intensive care unit
IMC: Intermediate care
OPS: Operation and procedure code
PCT: Procalcitonin
ROC: Receiver operating characteristic
ULN: Upper limit of normal
