# Supplementary material for: Identification of early predictors for infected necrosis in acute pancreatitis
Source: BMC Gastroenterol. 2022 Sep 3;22:405. doi: 10.1186/s12876-022-02490-9 (PMC9440524; doi:10.1186/s12876-022-02490-9)
Supplement: Supplementary file 1 — Additional file 1. Table S1. Model development with complete list of parameters. [file 12876_2022_2490_MOESM1_ESM.docx]

**Additional file 1: Table S1** Extent of necrosis stratified by infection status

|  | Infected necrosis  (n = 59) | Sterile necrosis  (n = 30) | p-value^a^ |
| --- | --- | --- | --- |
|  |  |  | 0.426 |
| < 30% | 35 (59.3) | 19 (63.3) |  |
| 30% - 50% | 14 (23.7) | 9 (30.0) |  |
| > 50% | 10 (16.9) | 2 (6.7) |  |

Signiﬁcant differences between groups were tested using Fisher’s exact test
