# Supplementary material for: Identification of early predictors for infected necrosis in acute pancreatitis
Source: BMC Gastroenterol. 2022 Sep 3;22:405. doi: 10.1186/s12876-022-02490-9 (PMC9440524; doi:10.1186/s12876-022-02490-9)
Supplement: Supplementary file 2 — Additional file 2. Table S2. Microbial composition of infected necrosis (n = 59). [file 12876_2022_2490_MOESM2_ESM.docx]

**Additional file 2: Table S2** Microbial composition of infected necrosis (n=59)

| **Isolated pathogens from necrosis** | **Number of patients (%)** |
| --- | --- |
| **Gram-positive bacteria**  Enterococcus faecialis  Enterococcus faecium  Staphylococcus epidermidis  Staphylococcus aureus  Staphylococcus haemolyticus  Staphylococcus xylosus  Streptococcus spp. | **43 (72.88)**  7 (11.86)  28 (47.46)  8 (13.56)  7 (11.86)  3 (5.08)  3 (5.08)  4 (6.78) |
| **Gram-negative bacteria**  Escherichia coli  Pseudomonas aeruginosa  Enterobacter cloacae  Bacterioides spp.  Citrobacter freundii  Klebsiella  Stenotrophomonas maltophilia  Enterobacter aerogenes | **30 (50.85)**  9 (15.25)  8 (13.56)  4 (6.78)  3 (5.08)  1 (1.69)  6 (10.17)  3 (5.08)  1 (1.69) |
| **Candida spp.** | **26 (44.07)** |
| C. albicans | 22 (37.29) |
| C. glabrata/C. kefyr  C. tropicalis | 3 (5.08)  1 (1.69) |
