# Supplementary material for: Identification of early predictors for infected necrosis in acute pancreatitis
Source: BMC Gastroenterol. 2022 Sep 3;22:405. doi: 10.1186/s12876-022-02490-9 (PMC9440524; doi:10.1186/s12876-022-02490-9)
Supplement: Supplementary file 3 — Additional file 3. Table S3. Model development with complete list of parameters. [file 12876_2022_2490_MOESM3_ESM.docx]

**Additional file 3: Table S3** Model development with complete list of parameters

| Model | Cox & Snell R2 | Nagelkerke’s R2 | p-value |
| --- | --- | --- | --- |
| **Univariate Models** |  |  |  |
| Creatinine | 0.162 | 0.225 | < 0.001 |
| C-reactive protein | 0.159 | 0.220 | < 0.001 |
| Albumin | 0.116 | 0.162 | 0.002 |
| Lactate dehydrogenase | 0.108 | 0.155 | 0.007 |
| Urea | 0.107 | 0.149 | 0.002 |
| Total leukocyte count | 0.067 | 0.093 | 0.013 |
| Prothrombin time | 0.060 | 0.084 | 0.019 |
| Bilirubin | 0.056 | 0.078 | 0.024 |
| Calcium | 0.054 | 0.075 | 0.027 |
| Acute on chronic pancreatitis | 0.050 | 0.069 | 0.033 |
| Alcoholic etiology | 0.044 | 0.061 | 0.045 |
| **2 Parameter Multivariate Models** |  |  |  |
| Creatinine + C-reactive protein | 0.244 | 0.338 | < 0.001 |
| Creatinine + Albumin | 0.275 | 0.385 | < 0.001 |
| Creatinine + Lactate dehydrogenase | 0.173 | 0.247 | 0.003 |
| Creatinine + Urea | 0.162 | 0.226 | < 0.001 |
| Creatinine + Total leukocyte count | 0.195 | 0.270 | < 0.001 |
| Creatinine + Prothrombin time | 0.184 | 0.254 | < 0.001 |
| Creatinine + Bilirubin | 0.190 | 0.265 | < 0.001 |
| Creatinine + Calcium | 0.175 | 0.244 | < 0.001 |
| Creatinine + Acute on chronic pancreatitis | 0.177 | 0.245 | < 0.001 |
| Creatinine + Alcoholic etiology | 0.194 | 0.269 | < 0.001 |
| **3 Parameter Multivariate Models** |  |  |  |
| Creatinine + Albumin + C-reactive protein | 0.305 | 0.425 | < 0.001 |
| Creatinine + Albumin + Lactate dehydrogenase | 0.265 | 0.383 | 0.001 |
| Creatinine + Albumin + Urea | 0.281 | 0.393 | < 0.001 |
| Creatinine + Albumin + Total leukocyte count | 0.300 | 0.419 | < 0.001 |
| Creatinine + Albumin + Prothrombin time | 0.277 | 0.387 | < 0.001 |
| Creatinine + Albumin + Bilirubin | 0.299 | 0.421 | < 0.001 |
| Creatinine + Albumin + Calcium | 0.278 | 0.392 | < 0.001 |
| Creatinine + Albumin + Acute on chronic pancreatitis | 0.283 | 0.396 | < 0.001 |
| Creatinine + Albumin + Alcoholic etiology | 0.327 | 0.458 | < 0.001 |
| **4 Parameter Multivariate Models** |  |  |  |
| Creatinine + Albumin + Alcoholic etiology + C-reactive protein | 0.360 | 0.502 | < 0.001 |
| Creatinine + Albumin + Alcoholic etiology + Lactate dehydrogenase | 0.328 | 0.476 | < 0.001 |
| Creatinine + Albumin + Alcoholic etiology + Urea | 0.341 | 0.477 | < 0.001 |
| Creatinine + Albumin + Alcoholic etiology + Total leukocyte count | 0.351 | 0.491 | < 0.001 |
| Creatinine + Albumin + Alcoholic etiology + Prothrombin time | 0.330 | 0.462 | < 0.001 |
| Creatinine + Albumin + Alcoholic etiology + Bilirubin | 0.331 | 0.466 | < 0.001 |
| Creatinine + Albumin + Alcoholic etiology + Calcium | 0.326 | 0.459 | < 0.001 |
| Creatinine + Albumin + Alcoholic etiology + Acute on chronic pancreatitis | 0.329 | 0.461 | < 0.001 |
| **5 Parameter Multivariate Models** |  |  |  |
| Creatinine + Albumin + Alcoholic etiology + C-reactive protein + Lactate dehydrogenase | 0.363 | 0.526 | < 0.001 |
| Creatinine + Albumin + Alcoholic etiology + C-reactive protein + Urea | 0.373 | 0.519 | < 0.001 |
| Creatinine + Albumin + Alcoholic etiology + C-reactive protein + Total leukocyte count | 0.367 | 0.512 | < 0.001 |
| Creatinine + Albumin + Alcoholic etiology + C-reactive protein + Prothrombin time | 0.362 | 0.505 | < 0.001 |
| Creatinine + Albumin + Alcoholic etiology + C-reactive protein + Bilirubin | 0.366 | 0.513 | < 0.001 |
| Creatinine + Albumin + Alcoholic etiology + C-reactive protein + Calcium | 0.367 | 0.514 | < 0.001 |
| Creatinine + Albumin + Alcoholic etiology + C-reactive protein + Acute on chronic pancreatitis | 0.361 | 0.504 | < 0.001 |
